# Prevalence and predictors of posttraumatic stress disorder, depression and anxiety among hospitalized patients with coronavirus disease 2019 in China

**DOI:** 10.1186/s12888-021-03076-7

**Published:** 2021-02-08

**Authors:** Yaru Chen, Xin Huang, Chengyuan Zhang, Yuanyuan An, Yiming Liang, Yufang Yang, Zhengkui Liu

**Affiliations:** 1 CAS Key Laboratory of Mental Health, Institute of Psychology, Chinese Academy of Sciences, 16 Lincui Road, Chaoyang District, Beijing, 100101 China; 2 Department of Psychology, University of Chinese Academy of Sciences, Beijing, China; 3 Department of Respiratory and Critical Care Medicine, East District of the First Affiliated Hospital of Anhui Medical University (Feidong People’s Hospital), Feidong, China; 4 School of Psychology, Nanjing Normal University, Nanjing, China

**Keywords:** COVID-19, Hospitalized patients, Posttraumatic stress disorder (PTSD), Depression, Anxiety

## Abstract

**Background:**

Coronavirus disease 2019 (COVID-19) has affected more than 5 million people around the world and killed more than 300,000 people; thus, it has become a global public health emergency. Our objective was to investigate the mental health of hospitalized patients diagnosed with COVID-19.

**Methods:**

The PTSD checklist for DSM-5 (PCL-5), Patient Health Questionnaire (PHQ-9), Generalized Anxiety Disorder Scale (GAD-7), Trauma Exposure Scale, abbreviated version of the Connor–Davidson Resilience Scale (CD-RISC-10), Perceived Social Support Scale (PSSS) and Demographic Questionnaire were used to examine posttraumatic stress disorder (PTSD), depression, anxiety, trauma exposure, resilience and perceived social support among 898 patients who were hospitalized after being diagnosed with COVID-19 in China. The data were analyzed with *t* tests, one-way ANOVA and multivariable logistic regression analysis.

**Results:**

The results showed that the prevalence of PTSD, depression and anxiety was 13.2, 21.0 and 16.4%, respectively. Hospitalized patients who were more impacted by negative news reports, had greater exposure to traumatic experiences, and had lower levels of perceived social support reported higher PTSD, depression and anxiety.

**Conclusions:**

Effective professional mental health services should be designed to support the psychological wellbeing of hospitalized patients, especially those who have severe disease, are strongly affected by negative news and have high levels of exposure to trauma.

## Background

Coronavirus disease 2019 (COVID-19) has affected more than 5 million people and caused more than 300,000 deaths worldwide in just a few months. The World Health Organization (WHO) declared the outbreak a pandemic in March 2020 and stated that the world was facing a global health crisis [1]. To date, most studies have focused on mental health problems in medical staff and the general population [2–4], and research involving hospitalized patients with COVID-19 has focused on treatments for the disease [5]. As observed during the epidemics of severe acute respiratory syndrome (SARS), novel influenza A (H1N1), Ebola virus disease (EVD) and Middle East respiratory syndrome (MERS), patients experience serious psychological problems in the acute phase of the disease and in the long term after an epidemic [6–10]. Therefore, the mental health problems experienced by hospitalized patients with COVID-19 must be detected and treated promptly. This issue deserves global attention. However, no detailed study on the mental health status of patients during the pandemic has been conducted to date.

According to previous studies, the onset of a sudden and immediately life-threatening illness can lead to posttraumatic stress disorder (PTSD) [11]. The psychological pressure on COVID-19 patients due to isolation treatment and other reasons may be far greater than that of the general public, and the situations they face during hospitalization also produce potential trauma [12]. According to past experiences of epidemics, more than 40% of SARS survivors experienced posttraumatic stress symptoms at some time during the outbreak [10, 13]. In addition to PTSD, depression and anxiety are common mental health problems in patients with infectious diseases. Kim et al. [7] investigated patients with suspected and confirmed MERS who were isolated in hospitals and found that the prevalence of depression was 40.7%. Wu et al. [14] reported that 14% of SARS patients reported anxiety 1 month after they were discharged from the hospital. Consequently, PTSD, depression and anxiety in patients during the COVID-19 outbreak should be afforded more attention.

Moreover, PTSD commonly co-occurs with other psychiatric disorders over a range of populations and trauma types [15]. Among subjects with PTSD, the rates of comorbid depression and anxiety range from 21 to 94% and from 39 to 97%, respectively [16]. Previous studies showed that 10 to 35% of SARS survivors reported symptoms of depression, anxiety or both during the early recovery phase [14, 17]. Compared with PTSD alone, PTSD combined with other psychiatric diseases is more difficult to treat [18]. Therefore, it is very important to identify psychiatric comorbidities of PTSD in hospitalized patients with COVID-19 to enable the implementation of interventions as soon as possible. In addition, it is critical to determine the factors that are predictive of these comorbidities.

Evidence has shown that when PTSD and other psychiatric disorders co-occur in populations who have experienced trauma, a combined stress model with shared vulnerabilities and similar risk factors might be involved [18]. Therefore, it is important to identify risk factors for not only the psychiatric disorders of interest but also comorbid disorders. North et al. [19] systematically analyzed the factors predictive of mental health problems in survivors of 10 disasters. Their review noted that the research findings to date collectively indicate that the risk of mental health problems following disasters is generally associated with female sex, young age, minority ethnicity, lower socioeconomic status, higher education level, marital status (married for women and unmarried for men), predisaster psychiatric illness, greater exposure to the disaster, and lack of perceived and actual social support. Focusing on the differences in demographic variables between individuals with relatively high and low scores for PTSD, depression and anxiety helps to predict the populations at high risk for these disorders. This study focuses on sex, age, educational background and socioeconomic status. The degree of trauma exposure is a direct inducer of the posttraumatic psychological response [20]. Trauma exposure refers to objective factors such as the degree of injury to oneself, relatives and friends and subjective experiences such as worrying about the safety of oneself and important others [21]. Studies have found that media exposure during critical public events may cause additional psychological trauma and anxiety, indicating that the vicarious traumatization effect may play an important role in the development of psychological disorders [22, 23]. Hence, exposure to news reports related to the pandemic is considered a candidate risk factor.

In addition, resilience and perceived social support are considered protective factors against an adverse psychological response. Resilience is the ability to maintain relatively stable, healthy levels of psychological and physical functioning in the context of adversity, stress or trauma [24]. After patients have been diagnosed with COVID-19, resilience enables them to cope with the stressful event through the interaction of external resources and internal potential [25]. Perceived social support has been identified as a strong external resource [26]. According to Cohen and Wills [27] and Goyne and Downey [28], there are two models to explain the role of social support in the posttraumatic psychological response. The direct effect model suggests that social support can directly promote individual posttraumatic adaptation by improving an individual’s healthy behavior and reducing the negative psychological response after the traumatic event. The buffering effect model of social support holds that providing resources for individuals to use when coping with traumatic events helps to alleviate the negative impact of these events on individuals.

This study was performed to provide information about the prevalence rates of PTSD, depression, and anxiety among COVID-19 patients in China and to explore the associated risk factors. The related factors examined were demographic variables, trauma exposure and psychosocial variables.

## Methods

### Participants

Starting in March 2020, we recruited patients who had been hospitalized for COVID-19 at the end of January and early February in China, primarily from Wuhan in Hubei Province. The patients began to return to the hospital for reexamination in the middle of March.

A total of 909 hospitalized patients with COVID-19 were recruited as of May 2020. After eliminating 5 participants aged under 16 years old, 1 participant who reported a history of psychiatric disease and 5 participants who were invalidated due to incomplete or careless answers, the final number of participants was 898. The sociodemographic information is shown in Table 1. The mean age of the participants was 39.40 years (*SD* = 14.05), with a range from 16 to 92 years. Of the participants, 123 (13.7%) were younger than 20 years old, 336 (37.4%) were between 21 and 40, 370 (41.2%) were between 41 and 60, and 62 (6.9%) were over 60 years. A total of 382 (42.5%) participants were male. In terms of educational background, 127 (14.1%) participants had a diploma from a primary or secondary school, 204 (22.7%) participants had a diploma from high school or technical secondary school, 532 (59.2%) participants had a diploma from a university or technical college, and 35 (3.9%) participants had a graduate degree or above. In terms of subjective socioeconomic status, 96 (10.7%) participants were at a low level, 316 (35.2%) participants were at a below-average level, 449 (50.0%) participants were at an average level, and 37 (4.1%) participants were at an above-average level or high level.

**Table 1:** Demographic differences among patients with PTSD, depression and anxiety (*n* = 898)

	Respondents			PTSD				Depression				Anxiety		
	N	%	*M*	*SD*	*F/t*	*p*-value	*M*	*SD*	*F/t*	*p*-value	*M*	*SD*	*F/t*	*p*-value
Overall	898	100	15.88	14.62			6.72	5.62			4.71	5.12		
Sex														
Male	382	42.5	15.06	14.46	−1.44	.152	5.91	5.53	−1.66	.097	4.31	5.01	−2.04	.041
Female	516	57.5	16.48	14.72			6.54	5.67			5.01	5.19		
Age, years														
≤ 20	123	13.7	11.81	14.50	4.84	.002	5.54	5.19	0.91	.435	3.06	4.58	5.34	.001
21–40	336	37.4	15.64	15.40			6.45	5.95			5.01	5.40		
41–60	370	41.2	16.98	13.84			6.28	5.44			4.89	4.94		
≥ 61	62	6.9	18.90	14.62			6.69	5.62			5.50	5.24		
Educational background														
Primary and secondary school	127	14.1	19.49	15.58	4.17	.006	6.64	5.73	0.33	.801	5.63	5.45	3.63	.013
High school or technical school	204	22.7	16.75	13.33			6.27	5.32			5.15	4.90		
University or college	532	59.2	14.64	14.69			6.15	5.70			4.26	5.01		
Postgraduate or above	35	3.9	16.54	15.11			6.71	5.85			5.66	6.25		
Socioeconomic status														
Low	96	10.7	19.51	16.99	3.32	.019	7.45	6.47	3.62	.013	6.27	5.93	4.45	.004
Below-average	316	35.2	16.58	14.76			6.68	5.83			4.87	5.20		
Average	449	50.0	14.66	13.84			5.69	5.21			4.24	4.77		
Above-average to high	37	4.1	15.14	14.60			6.62	5.73			5.08	5.62		

### Procedures

From March 22, 2020, to May 24, 2020, medical and psychological health care workers in hospitals for COVID-19 introduced this investigation to patients with COVID-19. Due to the pandemic disease prevention and control measures, the questionnaire was completed online in the WeChat application after informed consent was obtained. The method of completing the questionnaire was very simple. The participants clicked the link shared by the psychological health care workers to access the questionnaire. After answering all the questions, the patients then clicked the submit button. If the participants had any operational questions or difficulties completing the questionnaire, the psychological health care workers provided guidance. The patients were notified that online and in-person psychological counseling services were available if needed after the survey.

The study design and procedures were approved by the ethics review committee of the Institute of Psychology, Chinese Academy of Sciences (Protocol name: Evaluation and intervention of mental health of people affected by COVID-19).

### Measures

#### PTSD checklist for DSM-5 (PCL-5)

PTSD was assessed with the PTSD checklist for DSM-5 (PCL-5), which was compiled by Weathers and colleagues [29], translated into Chinese and revised by Zhou, Wu and Zhen [30]. A total of 20 items rated on a 5-point scale ranging from 0 (never) to 4 (severe) were used to assess the frequency of symptoms after diagnosis with COVID-19. The participants were asked to report their symptoms in the last month. The PCL-5 is composed of four dimensions: intrusion, emotion alteration, avoidance, and hyperarousal. A total score is computed for each item, with higher scores indicating a higher degree of PTSD symptoms. Based on the clinical criteria [31], scores above 33 indicate probable posttraumatic stress symptoms. In the current study, Cronbach’s alpha for the scale was 0.962.

#### Patient health questionnaire (PHQ-9)

Depression was assessed with the Patient Health Questionnaire (PHQ-9), which was compiled by Kroenke, Spitzer, and Williams [32] and translated into Chinese and revised by Wang et al. [33]. A total of 9 items rated on a 4-point scale ranging from 0 (not at all) to 3 (almost every day) were used to assess the frequency of symptoms in the past 2 weeks. Based on the established criteria [34], scores of 10–14 indicate moderate depression, scores of 15–19 indicate severe depression, and scores of 20–27 indicate severe depression. In the current study, Cronbach’s alpha for the scale was 0.920.

#### Generalized anxiety disorder scale (GAD-7)

Anxiety was assessed with the Chinese version of the Generalized Anxiety Disorder Scale (GAD-7) [35]. A total of 7 items rated on a 4-point scale ranging from 0 (never) to 3 (almost every day) were used to assess the frequency of symptoms in the past 2 weeks. According to the established criteria [36], scores of 10–14 indicate moderate anxiety and scores of 15–21 indicate severe anxiety. In the current study, Cronbach’s alpha for the scale was 0.954.

#### Trauma exposure scale

Trauma exposure was assessed with the second part of the University of California at Los Angeles (UCLA) PTSD reaction index (UCLA PTSD-RI) [37]. A total of 13 items were used to assess the objective (e.g., Were you seriously ill?) and subjective (e.g., Did you feel very scared, like this was one of your scariest experiences ever?) features of exposure to trauma. These items were scored as present (1) or absent (0). A total score for the 13 items was used to indicate the degree of trauma exposure in the current study.

#### Abbreviated version of the Connor–Davidson resilience scale (CD-RISC-10)

Resilience was assessed with the abbreviated version of the Connor–Davidson Resilience Scale (CD-RISC-10), which was compiled by Connor and Davidson [38] and revised by Wang et al. [39]. A total of 10 items rated on a 5-point scale ranging from 0 (not true at all) to 4 (true nearly all the time) were used to assess agreement with statements of psychological regulation in the last month. Higher total scores indicate a high level of resilience. In the current study, Cronbach’s alpha for the scale was 0.969.

#### Perceived social support scale (PSSS)

Perceived social support was assessed with the Chinese version of the Perceived Social Support Scale (PSSS) [40]. A total of 12 items were rated on a 7-point scale ranging from 1 (extremely disagree) to 7 (extremely agree). The scale is composed of two dimensions: family endogenous support and family exogenous support. A total score is computed for each dimension, with higher scores indicating a higher level of perceived social support. In the current study, Cronbach’s alpha for the full scale was 0.954.

#### Sociodemographic information

Participants were asked to report their sex, age, educational background, socioeconomic status and the impact of news reports. One item was used to assess subjective socioeconomic status (e.g., How do you think the living standard of your family compares to that of the whole country?) ranging from 1 (low) to 4 (above-average to high). Two items were used to assess the negative impact of news reports (e.g., To what extent have you been negatively affected by news coverage of COVID-19?) and the positive impact of news reports (e.g., To what extent have you been positively affected by news coverage of COVID-19?) ranging from 1 (almost none) to 6 (almost all).

### Data analysis

Data were analyzed with SPSS 26.0. Independent sample *t* tests and one-way ANOVA were used to examine differences in sociodemographic information among patients with different scores for PTSD, depression and anxiety. Multivariate logistic regression analyses were performed to identify the risk factors for PTSD, depression and anxiety. All statistically significant associated variables in Table 1 were collectively entered into multivariable logistic regression analyses. Odds ratios (ORs) with 95% confidence intervals (95% CIs) were calculated to measure the strength of association. All statistical tests were 2-tailed, and a *p*-value of less than .05 considered statistically significant.

## Results

### Prevalence of PTSD, depression and anxiety and the associated demographics

Using a cutoff score of 33, 119 (13.2%) patients had probable posttraumatic stress symptoms. Using a cutoff score of 10, the prevalence of depression was 21.0%, and the prevalence of moderate, severe and very severe depression was 11.5, 6.3 and 3.2%, respectively. Using a cutoff score of 10, the prevalence of anxiety was 16.4%, and the prevalence of moderate and severe anxiety was 10.9 and 5.5%, respectively. Among the participants with PTSD, 77.3% had comorbid depression and 65.5% had comorbid anxiety. Among the participants with depression, the proportion with comorbid anxiety was 65.6%. In total, 8.2% of the participants had three disorders.

### Effect of sociodemographics, trauma exposure, resilience and perceived social support on PTSD, depression and anxiety

The results of the multivariable logistic regression analysis are presented in Table 2. Hospitalized patients who were more impacted by negative news reports (OR = 1.72, 95% CI =1.37 ~ 2.15 for PTSD; OR = 1.69, 95% CI = 1.40 ~ 2.05 for depression; OR = 1.62, 95% CI = 1.32 ~ 1.99), had greater exposure to traumatic experiences (OR = 1.20, 95% CI =1.12 ~ 1.29 for PTSD; OR = 1.25, 95% CI = 1.18 ~ 1.33 for depression; OR = 1.24, 95% CI = 1.15 ~ 1.32), and had lower levels of perceived social support (OR = 0.96, 95% CI =0.95 ~ 1.00 for PTSD; OR = 0.97, 95% CI = 0.96 ~ 0.98 for depression; OR = 0.97, 95% CI = 0.95 ~ 0.98) were at a higher risk of PTSD, depression and anxiety. Hospitalized patients who had lower levels of resilience (OR = 0.98, 95% CI = 0.96 ~ 1.00) were at a higher risk of depression.

**Table 2:** Multivariable logistic regression analyses of possible factors associated with PTSD, depression and anxiety (*n* = 898)

	PTSD		Depression		Anxiety	
	OR (95% CI)	*p*-value	OR (95% CI)	*p*-value	OR (95% CI)	*p*-value
Sex						
Male	1.04 (0.66, 1.61)	.881	1.04 (0.71, 1.52)		1.01 (0.67, 1.53)	.955
Female	1		1		1	
Age, years						
≤ 20	1.06 (0.37, 3.03)	.912	1.57 (0.61, 4.05)	.355	0.65 (0.23, 1.81)	.405
21–40	0.79 (0.33, 1.88)	.592	1.31 (0.58, 2.93)	.516	0.86 (0.39, 1.93)	.720
41–60	0.57 (0.25, 1.30)	.182	0.96 (0.45, 2.07)	.915	0.56 (0.26, 1.20)	.134
≥ 61	1		1			
Educational background						
Primary and secondary school	0.95 (0.28, 3.31)	.941	0.74 (0.26, 2.12)	.573	0.49 (0.17, 1.45)	.199
High school or technical school	0.81 (0.25, 2.59)	.721	0.73 (0.27, 1.92)	.519	0.56 (0.21, 1.79)	.249
University or college	1.02 (0.34, 3.03)	.972	0.94 (0.38, 2.35)	.902	0.57 (0.22, 1.51)	.232
Postgraduate or above	1		1		1	
Socioeconomic status						
Low	1.96 (0.53, 7.31)	.314	0.93 (0.33, 2.61)	.893	1.23 (0.42, 3.58)	.711
Below-average	1.52 (0.46, 5.18)	.505	0.72 (0.28, 1.83)	.491	0.67 (0.25, 1.79)	.425
Average	1.31 (0.39, 4.43)	.666	0.54 (0.21, 1.34)	.181	0.57 (0.22, 1.51)	.262
Above-average to high	1		1		1	
Impact of negative news reports	1.72 (1.37, 2.15)	<.001	1.69 (1.40, 2.05)	<.001	1.62 (1.32, 1.99)	<.001
Impact of positive news reports	1.07 (0.86, 1.33)	.552	0.90 (0.74,1.09)	.270	1.05 (0.86, 1.29)	.635
Trauma exposure	1.20 (1.12,1.29)	<.001	1.25 (1.18, 1.33)	<.001	1.24 (1.15, 1.32)	<.001
Resilience	0.98 (0.95, 1.00)	.080	0.98 (0.96, 1.00)	.030	0.99 (0.96, 1.01)	.220
Perceived social support	0.96 (0.95, 0.98)	<.001	0.97 (0.96, 0.98)	<.001	0.97 (0.95, 0.98)	<.001

Note: *OR* Odds ratio, *CI* Confidence interval

## Discussion

To the best of our knowledge, this is the first study to investigate the prevalence and predictors of psychiatric symptoms among hospitalized COVID-19 patients during the acute treatment period. The current study showed that the prevalence rates of PTSD, depression and anxiety among hospitalized patients with COVID-19 were high and that these disorders often co-occurred. Negative media reports, exposure to trauma and perceived social support were shared risk and protective factors of PTSD, anxiety and depression. Resilience was a protective factor only for depression.

### Prevalence of and differences in PTSD, depression and anxiety among hospitalized patients with COVID-19

In this study, the prevalence of PTSD, depression and anxiety among hospitalized patients with COVID-19 was 13.2, 21.0 and 16.4%, respectively. The prevalence of PTSD was lower than the prevalence in previous studies with patients with similar infectious diseases [10, 13] but higher than those in other groups, such as general residents and medical staff, during the COVID-19 pandemic [2–4]. The prevalence of depression and anxiety was higher than in previous studies on infectious diseases [7, 14] and studies involving residents [4] and medical workers [2] in Wuhan during the COVID-19 outbreak. The results indicate that hospitalized patients who have contracted COVID-19 may have the most serious mental health problems during this pandemic. The possible causes are related to the virus itself, the use of corticosteroids or hydroxychloroquine, or pandemic-related stress. First, the virus that causes COVID-19 might infect the brain or trigger immune responses that have additional adverse effects on brain function and mental health in patients with COVID-19 [41, 42]. Second, corticosteroids may induce affective psychosis, and hydroxychloroquine use has been related to agitation, emotional lability and irritability [43, 44]. Third, the psychological pressure on COVID-19 patients due to isolation treatment may be far greater than that of medical workers and general residents [12]. Additionally, patients have a greater risk of death.

The study showed that PTSD, depression and anxiety are often comorbid. PTSD, depression, and generalized anxiety disorder (GAD) are all thought to stem from high levels of general distress in the acute stage of trauma and are likely to become a symptom network [45]. A network analysis study showed that symptoms of GAD (inability to relax) and PTSD (restricted or diminished positive emotion) were identified as key hub symptoms for the network of PTSD, depression and anxiety. Symptoms of depression and GAD are highly interrelated [46]. A previous study has shown that the association of anxiety and depression during the COVID-19 pandemic can be attributed to the strong connection among impaired motor skills, restlessness, and inability to relax [47]. During treatment for COVID-19, patients may develop multiple related psychiatric diseases that form a mutually influential symptom network. Therefore, mental illness intervention from the perspective of symptoms (e.g., impaired motor skills or inability to relax) rather than mental illness as a whole (e.g., PTSD or depression) may be a better choice for mental health interventions for patients. In particular, the relationship between symptoms in the context of COVID-19 needs to be studied further.

### Effects of trauma exposure, resilience and perceived social support on PTSD, depression and anxiety

This study found that negative media reports, trauma exposure and perceived social support were shared risk and protective factors of PTSD, anxiety and depression. Resilience was a protective factor only for depression. First, infection with an unknown virus for which there are no known treatments increases patients’ perception of a threat to their lives, which in turn can lead to the development of mental disorders. Second, as one aspect of trauma exposure, the degree of subjective fear plays an important role in inpatients’ susceptibility to mental health problems. Previous studies have reported that the subjective experience of fear is more strongly related to PTSD than objective exposure alone [48]. As evidenced in past epidemics [49], the perception of personal risk, rather than not only actual exposure, can confer considerable susceptibility to mental health disorders. Negative news reports and the severity of the disease can affect an individual’s subjective experience of fear. Sensational media reports may stimulate the subconscious perception of threat and induce fear [50], and fear has been shown to be associated with a considerable risk of the new onset and recurrence of mental health disorders [51]. Moreover, heightened distress responses to media reports of collective crises may have long-term physical health repercussions [22]. However, the positive impacts of media reports were not found to alleviate psychiatric disorders in this study.

In addition, resilience was a protective factor against depression, and perceived social support was a protective factor against PTSD, depression and anxiety. A possible reason is that external resources are more important than internal resources for patients infected with COVID-19. Social support can not only affect individuals directly by improving their health behaviors but can also affect individuals indirectly by providing resources to individuals [27, 28]. Resilience is a personality trait. In the face of life-threatening events such as COVID-19 infection, patients need to deal with their physical health rather than their mental health. However, according to previous studies, resilience plays an important role in the long-term mental health recovery of patients [11].

### Limitations and implications

There are some limitations of this study. First, our participants were a convenience sample mainly recruited from hospitals in Wuhan. It is uncertain whether our findings can be generalized to all patients during this pandemic. Second, the data used in this study came from patients’ self-reported questionnaires. In the future, clinical diagnoses of PTSD, depression and anxiety should be used. Third, this was a cross-sectional study. Directionality cannot be determined between the “predictors” (e.g., resilience, perceived social support) and mental health symptoms. The pattern and long-term clinical course of the psychiatric effects of COVID-19 should continue to be investigated, and there should be widespread awareness of the possible psychiatric impacts of a future reemergence of COVID-19. Future studies should exploit existing datasets and ongoing longitudinal studies in addition to establishing new cohorts to collect detailed information on psychological factors.

The findings of this study indicate that timely integrated mental health interventions are warranted for patients during the acute treatment phase of COVID-19. First, health authorities need to identify high-risk groups based on sociodemographic information and implement early psychological interventions. Females, elderly patients, patients with lower levels of education, and patients with lower socioeconomic status are target populations. Second, reducing news reports during the pandemic and disseminating accurate scientific information will help patients understand this novel virus, thereby alleviating their fear and reducing their perception of COVID-19 as a threat. Third, providing a supportive hospitalization environment will help foster resilience, enabling hospitalized patients to maintain relatively stable, healthy levels of psychological and physical functioning.

## Conclusions

The current study showed that the prevalence rates of PTSD, depression and anxiety among hospitalized patients with COVID-19 were high and that these disorders often co-occurred. Negative media reports, exposure to trauma and perceived social support were shared predictors of PTSD, anxiety and depression. Resilience was a protective factor only for depression. These findings indicate that timely integrated mental health interventions are warranted for patients during the acute treatment phase of COVID-19.

## Data Availability

The datasets used and analyzed during the current study are available from the corresponding author on reasonable request.
